# Cooperative induction of receptor tyrosine kinases contributes to adaptive MAPK drug resistance in melanoma through the PI3K pathway

**DOI:** 10.1002/cnr2.1736

**Published:** 2022-10-17

**Authors:** Tine Norman Alver, Karen‐Marie Heintz, Eivind Hovig, Sigurd L. Bøe

**Affiliations:** ^1^ Department of Tumor Biology, Institute for Cancer Research Oslo University Hospital Oslo Norway; ^2^ Department of Cancer Genetics, Institute for Cancer Research Oslo University Hospital Oslo Norway; ^3^ Faculty of Medicine University of Oslo Oslo Norway; ^4^ Department of Informatics University of Oslo Oslo Norway; ^5^ Department of Medical Biochemistry Oslo University Hospital, The Norwegian Radium Hospital Oslo Norway

**Keywords:** AXL, ERBB3, MITF, resistance, SOX10

## Abstract

Vemurafenib‐induced drug resistance in melanoma has been linked to receptor tyrosine kinase (RTK) upregulation. The MITF and SOX10 genes play roles as master regulators of melanocyte and melanoma development. Here, we aimed to explore the complex mechanisms behind the MITF/SOX10‐controlled RTK‐induced drug resistance in melanoma. To achieve this, we used a number of molecular techniques, including melanoma patient data from TCGA, vemurafenib‐resistant melanoma cell lines, and knock‐down studies. The melanoma cell lines were classified as proliferative or invasive based upon their MITF/AXL expression activity. We measured the change of expression activity for MITF/SOX10 and their receptor (AXL/ERBB3) and ligand (NRG1/GAS6) targets known to be involved in RTK‐induced drug resistance after vemurafenib treatment. We find that melanoma cell lines characterized as proliferative (high MITF low AXL), transform into an invasive (low MITF, high AXL) cell state after vemurafenib resistance, indicating novel feedback loops and advanced compensatory regulation mechanisms between the master regulators, receptors, and ligands involved in vemurafenib‐induced resistance. Together, our data disclose fine‐tuned mechanisms involved in RTK‐facilitated vemurafenib resistance that will be challenging to overcome by using single drug targeting strategies against melanoma.

## 1 INTRODUCTION

Malignant melanoma is an aggressive cancer with poor survival for patients with advanced disease. Besides immunotherapy, small molecule inhibitors selectively targeting the BRAF kinase such as vemurafenib, dabrafenib, and encorafenib have been successful in treating BRAF mutant melanoma. However, reactivation of the MAPK pathway as well as an increase in PI3K signaling is a major challenge for therapy response, due to both intrinsic and acquired resistance.^1^, ^2^, ^3^, ^4^, ^5^, ^6^, ^7^ In an effort to circumvent resistance mechanisms, targeting the downstream kinase MEK in combination with BRAF inhibitors has been introduced into the clinic. However, even though responses have improved when using combination treatments, resistance continues to be a major obstacle for efficient therapy responses.^6^, ^7^

In response to MAPK pathway inhibition, melanoma cells may undergo a transcriptional reprogramming event, where proliferative melanoma cells switch into a phenotypically distinct invasive cell population.^8^ The mechanism behind this process is incompletely understood. However, a study on the reprogramming phase in melanoma suggests MITF/SOX10 and AP1/TEAD as being the master regulators of the proliferative and the invasive transcriptome, respectively.^9^ Moreover, it has been shown that during BRAF inhibitor treatment melanoma cells can lose MITF expression and de‐differentiate, marking the transition to an invasive subpopulation of treatment‐resistant cells.^5^ Moreover, SOX 10 has been shown to have a paradoxical role in adaptive resistance.^10^ Increased transcriptional levels of SOX10 are suggested to desensitize BRAF‐mutant melanoma to MAPK inhibition, however, loss of SOX10 has also been shown to drive acquired resistance.^2^, ^11^ The lack of MITF and its upstream regulator SOX10 has also been shown to coincide with an upregulation of receptor tyrosine kinases (RTKs), including EGFR, ERBB3, and AXL, and in this way contributing to acquired resistance.^5^, ^12^, ^13^, ^14^ Analyses of the mechanisms of drug resistance have revealed redundancy among the many surface receptors. Understanding the role of SOX10 and MITF in RTK regulation may disclose how treatment‐induced transcriptional reprogramming could be utilized in the optimization of melanoma treatment strategies.

We therefore set out to investigate the role of the SOX10/MITF axis upon RTK regulation during the development of vemurafenib resistance. In a panel of melanoma cell lines, we monitored gene expression levels of ligands and receptors previously linked to the SOX10/MITF axis. We identified SOX10, MITF, ERBB3, and GAS6 as upregulated markers during early vemurafenib treatment, while EGFR, AXL, and NRG1 were upregulated after the establishment of vemurafenib resistance. Interestingly, we also found evidence for AXL/ERBB3 receptor redundancy, further demonstrating the challenges of various treatment strategies. Our study opens up for further elucidation of transcriptional reprogramming events during MAPK pathway inhibition that may contribute to stratifying the melanomas and serve as a tool for appropriate therapy selection.

## 2 RESULTS AND DISCUSSION

We set out to investigate whether the MITF/SOX10 axis affects ERBB3 and AXL receptors throughout melanoma progression and treatment, as RTKs are frequently identified as targets of negative feedback loops and involved in resistance mechanisms in melanoma.^14^, ^15^, ^16^, ^17^, ^18^ We chose to explore the ERBB3 and AXL receptors, as they have been shown to be involved with MITF in resistance towards small molecule inhibitors in melanoma.^3^, ^5^

### 2.1 Correlations in patient samples

**FIGURE 1:**
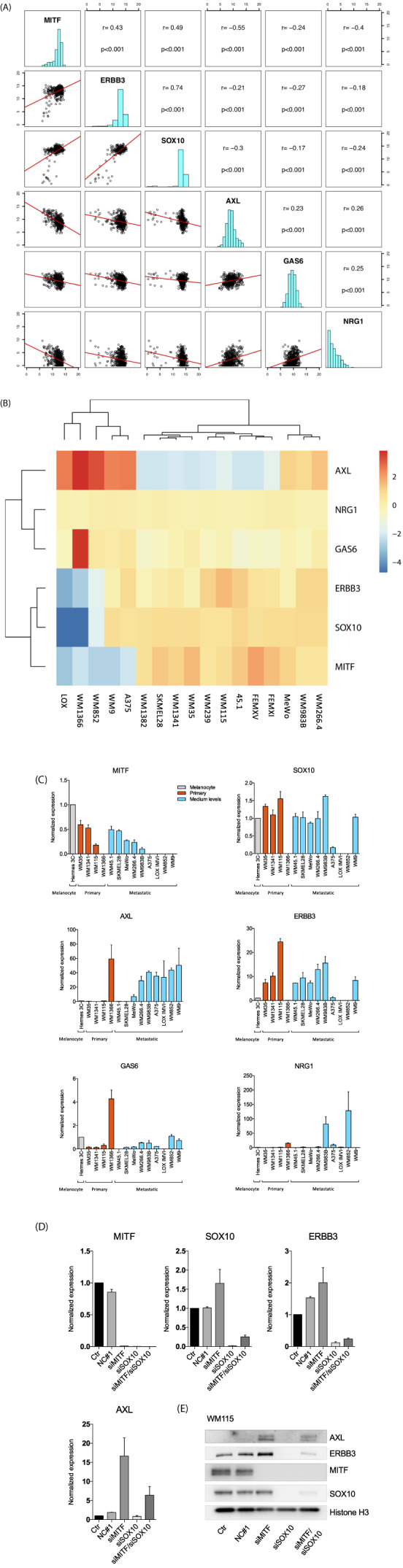
(A) Pairwise correlation plot of cutaneous skin cancer exon expression cases in TCGA (*n* = 473). IlluminaHiSeq‐defined RNA seq for the genes MITF, ERBB3, SOX10, AXL, GAS6, and NRG1. Both axes display log2 expression values. (B) Heat map showing AXL, NRG1; GAS6, ERBB3; SOX10 and MITF RNA expression levels indicated as high levels with yellow/orange/red color, and blue indicating low expression levels. LOX, WM1366, WM852, WM9, and A376 have high expression levels of AXL and low expression levels of MITF, SOX10, and ERBB3. FEMXI, FEMXV, WM45.1, WM115, WM239, WM35, WM1341, SKMEL28, and WM1382 have low AXL expression levels and high MITF, SOX10 and ERBB3 levels. WM266.4, WM983B, MeWo have high/medium levels of AXL, ERBB3, SOX10, and MITF. Expression values log2 + 1, (*n* = 1). (C) Basal expression levels of genes involved in AXL and ERBB3 pathway signaling. RT‐PCR was performed to evaluate mRNA levels of AXL, MITF, ERBB3, SOX10, GAS6, and NRG1 in a panel of melanoma cell lines normalized against immortalized melanocytes (Hermes 4C). Bars represent mean ± SEM (*n* = 3). (D) RT‐PCR data show mRNA levels of MITF, SOX10, ERBB3, and AXL after depletion of MITF and SOX10 alone or in combination. All experiments were normalized to untreated control cells. Results represent mean ± SEM (*n* = 2). (E) Representative Western blot showing the effect of MITF and SOX10 72 h siRNA treatment on protein levels of AXL, GAS6, ERBB3, and NRG1 (*n* = 3). Uncropped membranes are shown in the supplementary information.

We have previously examined 470 melanoma patient samples from the Cancer Genome Atlas (TCGA SKCM) to investigate correlations between MITF/SOX10, SOX10/ERBB3, and MITF/NRG1.^13^ We here expanded this examination by the inclusion of AXL and GAS6 correlations, and we observed an inverse correlation between AXL and MITF (*R* = −0.55, *p* = <.001), which is in agreement with results from previously published studies, in that melanomas lacking MITF exhibit high levels of AXL.^5^, ^19^ We also observed an inverse correlation between AXL and SOX10 (*R* = −0.3, *p* = <.001), and finally, a moderate inverse correlation was found between AXL and ERBB3 in patient samples (*R* = −0.21, *p* = <.001) (Figure 1A).

### 2.2 Correlations in melanoma cell lines

To investigate whether melanoma cell lines recapitulated the findings from the patient samples, we examined gene expression levels in a microarray format on a melanoma panel consisting of 17 cell lines spanning various mutational backgrounds and tumor stages. These cell lines represent the primary tumors WM115 (BRAFV600E), WM35 (BRAFV600E), WM1341 (BRAFV600E) and WM1366 (NRAS), and the metastatic cell lines A375 (BRAFV600E), WM9 (BRAFV600E), SKMEL28 (BRAFV600E), WM45.1 (BRAFV600E), WM983B (BRAFV600E), WM266.4 (BRAFV600E), WM239 (BRAFV600E), MeWo (NF1), FEMXI (HRAS), FEMXV (HRAS), WM1341 (triple wild type), and WM852 (NRAS). Clustering of the expression levels across the genes of interest appeared to be in agreement with the TCGA analysis, in that the cell lines A375, WM9, WM852, WM1366, and LOXIMVI show a clear inverse correlation between AXL and MITF, displaying low expression levels of MITF and high expression levels of AXL. WM852, WM1366, and LOX also display low expression levels of ERBB3 and SOX10, while A375 and WM9 show medium expression levels. FEMXI, FEMXV, 45.1, WM115, WM239, WM35, WM1341 and SKMEL28 cell lines display a clear inverse correlation, with high levels of MITF, SOX10 and ERBB3 and low levels of AXL. Finally, the MeWo, WM983B, and WM266.4 cell lines show high expression of all the genes of interest. Based on these findings, we classified our cell lines into three different groups based on their expression levels of AXL and ERBB3. The distinct population of high AXL expression and low MITF/SOX10/ERBB3 suggests that these cells are of the invasive phenotype, as previously proposed by Verfaille et al.^9^ The larger set of cell lines displayed a proliferative phenotype of high MITF/SOX10/ERBB3 and low AXL levels. Finally, a small cluster of cell lines, showing high levels of all four genes (MITF, SOX10, ERBB3, and AXL), represents a heterogenous phenotype, displaying both invasive and proliferative markers. (Figure 1B).

To further validate the results from the TCGA and gene expression data, we measured the basal RNA expression levels of NRG1, AXL, and GAS6, as well as utilizing our previously published values for SOX10, MITF, ERBB3.^13^ Our cell line panel consists of immortalized melanocytes and melanoma cell lines spanning various genetic backgrounds. We found that primary melanoma cell lines possessing the BRAFV600E mutation display low AXL and GAS6 levels as compared with melanocyte control cell line Hermes 3C. By contrast, WM1366, a vertical growth phase NRAS‐mutated cell line displayed high levels of AXL and GAS6 and low levels of ERBB3, SOX10, and MITF, showing an invasive marker (AXL) that implies a premetastatic state. In our metastatic cell lines, we observed the same inverse correlation, with the exception of in the WM9 cell line having high levels of SOX10, yet low levels of MITF. Our analyses suggest that the cell lines can be divided into three groups based on the expression levels of AXL and ERBB3. Cell lines with low MITF expression harbor high AXL levels, cell lines with high MITF and SOX10 levels have low AXL levels and high ERBB3 levels. Finally, cell lines with high SOX10 and low MITF levels display high levels of both ERBB3 and AXL, in agreement with the TCGA, expression array data, and Verfaillie et al.^9^ (Figure 1C).

In an attempt to further elucidate the interplay between MITF/SOX10 and ERBB3/AXL, we depleted SOX10 and MITF alone or in combination in the WM115 cell line, this cell line contains high levels of MITF, SOX10, and ERBB3, and low AXL expression. We found that MITF depletion alone increased both RNA and protein levels of AXL, ERBB3 and SOX10. Moreover, we found reduced RNA and protein levels of both MITF and ERBB3 following SOX10 depletion. AXL levels were not elevated following SOX10 depletion, while it did result in reduced MITF levels. This was surprising, as in theory, increased levels of AXL would be expected. Whether this is a general mechanism or exclusive to this cell line is currently unknown, and should be further explored. Depleting the combination of MITF and SOX10 demonstrated the expected downregulation of ERBB3,^20^ and upregulation of AXL, reflecting what we observed after siMITF treatment alone. Taken together, these results show that siRNA modulation of MITF/SOX10 directly affects ERBB3/AXL levels in melanoma cell lines (Figure 1D, E).

### 2.3 ERBB3 and AXL receptor redundancy

**FIGURE 2:**
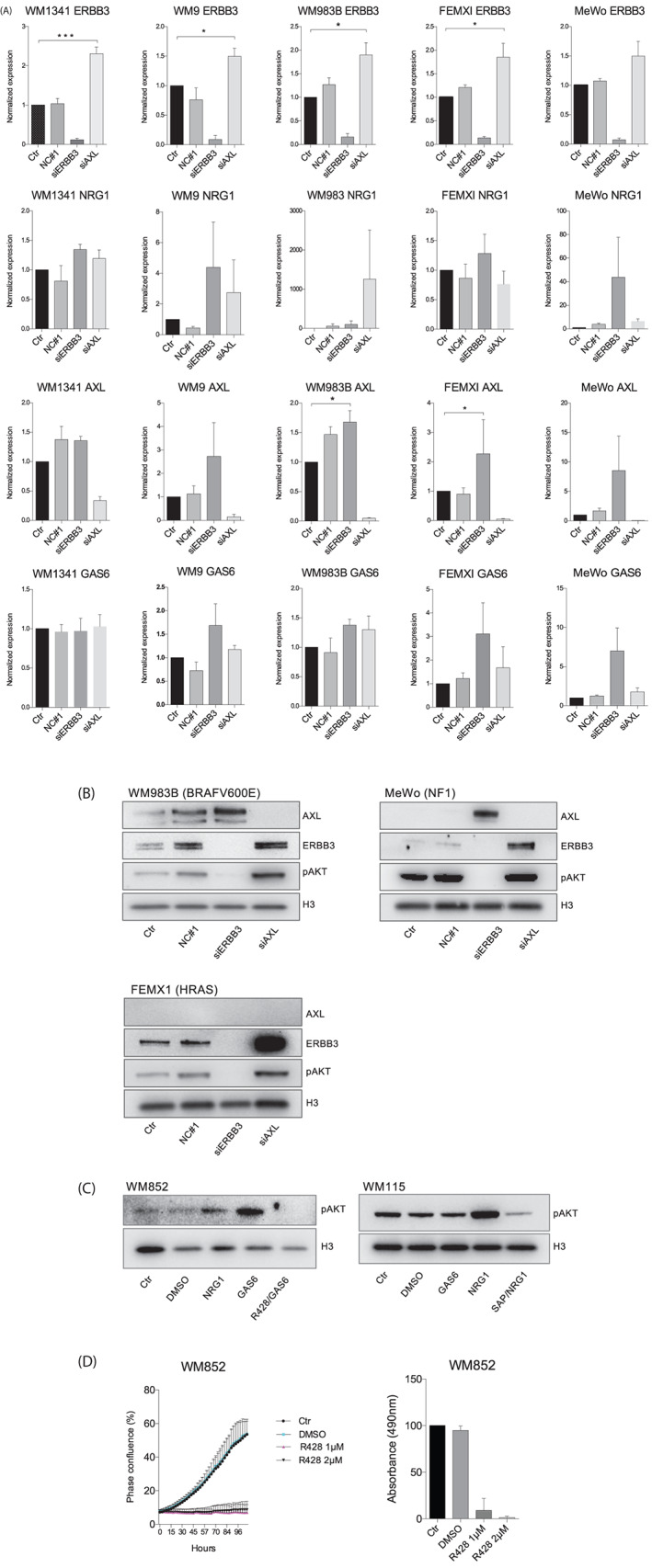
ERBB3 and AXL receptor redundancy. (A) RT‐PCR results showing the effect of siRNA against AXL and ERBB3 on the receptors and their respective ligands GAS6 and NRG1. WM1341, WM9, MeWo, WM983B, and FEMX‐1 cell lines were transfected with AXL and ERBB3 siRNA for 72 h before harvesting. All experiments were performed in triplicate and normalized to untreated control cells. Bars represent mean ± SEM (*n* = 3). *p < .05, ***p < .0001. (B) Representative western blots of the corresponding treatments. Uncropped membranes are shown in the supplementary information (*n* = 3). (C) Representative western blots of three independent experiments show the effect of 72 h small molecule inhibitor treatments using R428 (AXL inhibitor) in AXL high/ERBB3 low cell line WM852 and sapatinib (ERBB3 inhibitor) in AXL low/ERBB3 high cell line WM115. GAS6 (200 ng/ml) and NRG1 (10 ng/ml) ligands were added 15–20 min before cells were harvested for protein extraction. Uncropped membranes are shown in the supplementary information. (D) Growth curve demonstrating confluence over time during treatment with R428 (1 and 2 μM) measured by IncuCyte and subsequent cell viability measured by MTS (*n* = 3).

Our cell lines displayed two distinct phenotypic groups characterized by either high expression of AXL or high expression of ERBB3. We therefore wanted to investigate the possibility of receptor redundancy between ERBB3 and AXL. Five cell lines containing different mutational backgrounds were selected, WM1341, WM9, and WM983 represented BRAFV600E mutants, while MeWo represented NF1 mutations, and FEMXI represented HRAS‐mutant melanoma. We found that downregulation of AXL expression led to an increase in ERBB3 levels at both the RNA and protein levels in all cell lines tested. Interestingly, when knocking down AXL in the MeWo and WM983B cell lines, we observed an up‐regulation of ERBB3, indicating an apparent two‐way redundancy (Figure 2A, B). This is in line with recent results showing an AXL‐ and ERBB3‐redundancy involved in invadopodia formation in melanoma^21^ and is of importance within the development of novel treatment strategies against melanoma.

As both AXL and ERBB3 have been implicated in vemurafenib resistance and PI3K pathway activation we wanted to investigate whether the levels of receptors dictate this pathway activation, and whether signaling persists exclusively through one receptor, and not the other. We selected the two cell lines WM852 (AXL high/ERBB3 low) and WM115 (ERBB3 high/AXL low), representing opposite expression levels. We then treated WM852 with the AXL inhibitor R428 (BGB324/bemcentinib), and WM115 with the ERBB inhibitor sapatinib (AZD8931) for 72 h. 30 and 15 min prior to harvest, we added both the AXL ligand GAS6 and the ERBB3 ligand NRG1 to both cell lines, respectively. Western blots show that the addition of GAS6 activated pAKT in WM852, but not in WM115. By contrast, the addition of NRG1 activated pAKT in both cell lines, although just slightly in WM852. The reason for this might be that although the levels are low, ERBB3 is still expressed at low levels in the WM852 cell line. The addition of GAS6 to the R428‐treated cells did not lead to activation of pAKT, demonstrating the efficacy of the inhibitor in this cell line. Moreover, the addition of NRG1 to the sapatinib‐treated WM115 cells reduced pAKT activation significantly as compared with control (Figure 2C).

We next asked whether this AXL high, MITF/SOX10/ERBB3 low, and NRAS‐mutated cell line could be treated with AXL inhibitor monotherapy. Using MTS and IncuCyte measures as readout, we found that the WM852 cell line stopped proliferating at relatively low doses (1 and 2 μM), suggesting that the AXL inhibitor R428 alone may abrogate growth in these cells (Figure 2D). These results are in line with a previous study, where it was demonstrated that the AXL receptor inhibitor amuvatinib had a cytotoxic effect against NRAS mutated melanoma.^22^

### 2.4 Receptor redundancy during vemurafenib treatment

**FIGURE 3:**
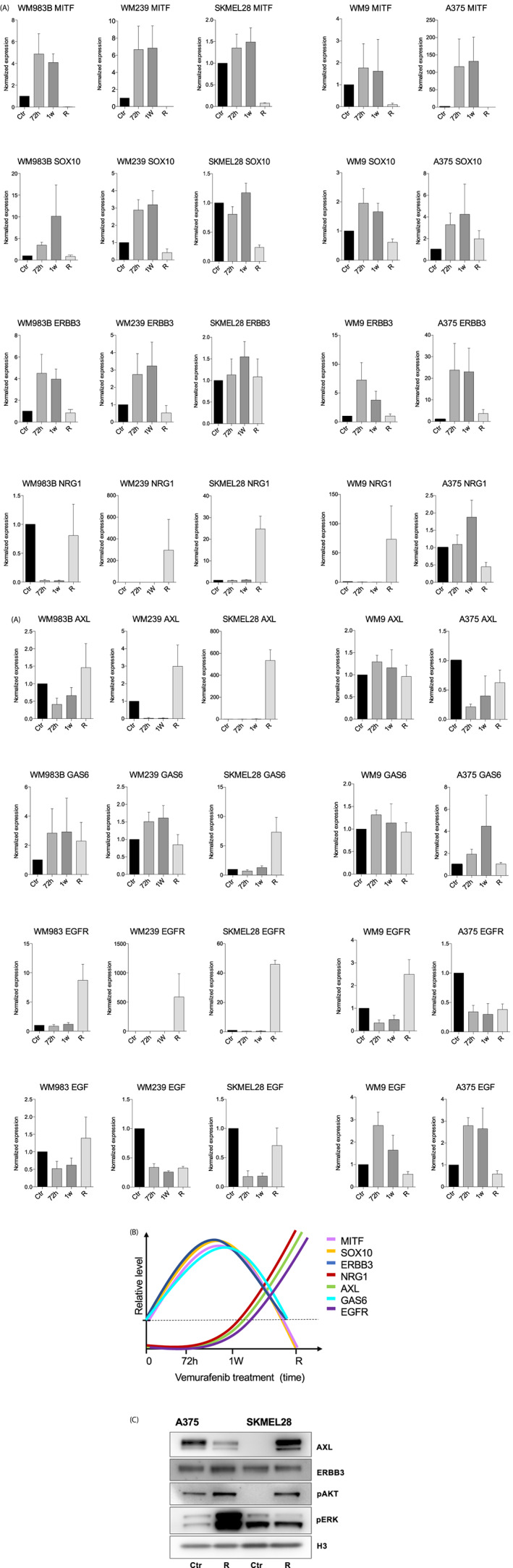
ERBB3 and AXL receptor redundancy during vemurafenib treatment. (A) RT‐PCR results showing the effect of vemurafenib treatment in WM983B, WM239, SKMEL28, WM9, and A375 cell lines. Treatment was started at a 1 μM concentration and increased with 0.5 μM at regular intervals until cells proliferated at a 3 μM concentration. All experiments were performed in triplicate and normalized to untreated control cells. Bars represent mean ± SEM (*n* = 3). (B) Model graph illustrating the adaptive behavior of genes of interest during MAPK inhibition in WM239, WM983B, and SKMEL28 cell lines. (C) Representative western blot showing protein levels of AXL, ERBB3, pAKT, and pERK in resistant A375 and SKMEL28 compared with untreated control (*n* = 3). Uncropped membranes can be found in the supplementary information.

To further investigate the consequences of AXL and ERBB3 receptor redundancy, we wanted to study the dynamics of the receptors in our cell lines in relation to the acquisition of resistance against BRAF inhibition. We treated the BRAFV600E mutated melanoma cell lines WM983, WM239, SKMEL28, WM9, and A375 with an increasing dose of the BRAF inhibitor vemurafenib. We considered the cells resistant when they proliferated at a 3 μM vemurafenib concentration, and we collected mRNA after 72 h, 1 week, and after the acquisition of resistance. RT‐PCR results showed MITF and SOX10 upregulation at 72 h and 1 week following treatment in all five cell lines, with a loss of both MITF and SOX10 expression after resistance had been attained. Moreover, we found that ERBB3 was upregulated at both 72 h and 1 week following vemurafenib treatment in WM983B, WM239, WM9, and A375, in agreement with previously published work.^3^, ^13^ This is also supported by our previous work, where we measured ERBB3 levels after 2 weeks of vemurafenib treatment in WM983B and SKMEL28 at the protein level.^13^ However, ERBB3 expression was comparable to normal levels in resistant cells. This seems to be compensated for by an increase of the ERBB3 ligand NRG1 in the resistant cells, which implies sustained signaling through the NRG1‐ERBB3‐PI3K pathway. NRG1 has previously been suggested to promote compensatory signaling through ERBB3 signaling in melanoma and colorectal cancer after BRAF inhibitor treatment.^23^, ^24^ Furthermore, the AXL ligand GAS6 was slightly upregulated after 72 h, and after 1 week, while the levels were reduced at the time of resistance with the exception of in the SKMEL28 cell line. Interestingly, AXL receptor levels were upregulated following resistance establishment in the AXL low/medium cell lines WM983B, WM239, and SKMEL28, while the AXL high and MITF low cell lines WM9 and A375 retain about the same transcription level of the factors after resistance as those of untreated controls. In contrast to NRG1‐ERBB3 signaling, AXL upregulation may occur without an apparent GAS6 dysregulation in patient samples. In addition, AXL bypass signaling acts independently of GAS6 in approximately half of drug resistant lung cancer cell lines examined.^25^ Furthermore, we included the RTK receptor EGFR, as it has been reported to confer resistance to MAPK inhibitors,^12^ and has also been shown to regulate cell invasion signaling via AXL in glioblastoma cells.^26^ In agreement with these studies, our results show that EGFR follows AXL expression during treatment, and eventually resistance, in our melanoma cell lines (Figure 3A). Figure 3B illustrates the adaptive behavior of the transcription factors, RTKs, and their ligands during vemurafenib treatment in SKMEL28, WM983B, and WM239 cell lines.

Interestingly, Western blots of the AXL high/MITF low cell line A375 show a small pAKT increase, reduced AXL levels, and an extensive pERK level increase, suggesting that the A375 cell line mainly obtains resistance through increased MAPK pathway signaling. By contrast, the AXL low cell line SKMEL28 displays unchanged pERK levels, increased AXL levels, and increased pAKT levels, implying PI3K‐induced resistance (Figure 3C).

**FIGURE 4:**
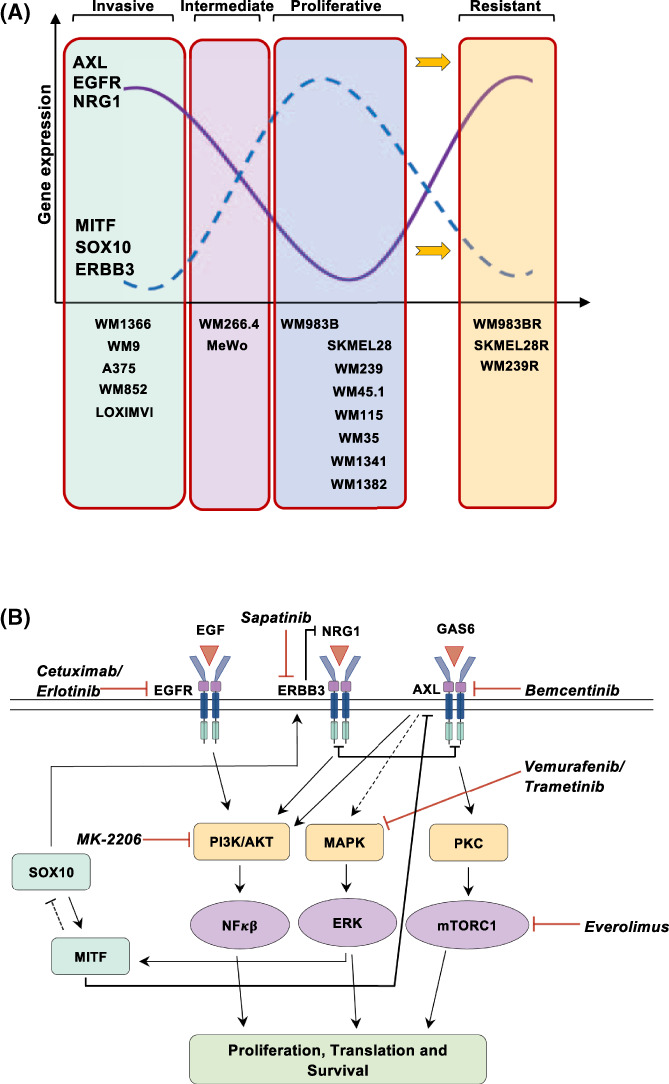
(A) Stratification of cell lines based on their transcriptional signature. The WM1366 cell line is thought to be classified as primary melanoma. However, stratifying it according to the transcriptional signature indicated in our results would suggest that this cell line is on the verge of becoming metastatic/invasive. Moreover, WM9, A375, LOXIMVI, and WM852 were stratified as invasive. MeWo and WM266.4 were stratified as being intermediate as they display medium‐to‐high levels of both invasive and proliferative signatures. Classified as of the Proliferative phenotype were WM983, SKMEL28, WM45.1, WM239, WM35, WM115, WM1341, and WM1382 cell lines. Finally, we included the transcriptional signature in the three treatment‐resistant cell lines WM983BR, SKMEL28R, and WM239R resembling the invasive phenotype displaying Low levels of MITF, SOX10 and ERBB3 and high levels of AXL. B. Illustration of proposed signaling pathways involved in vemurafenib‐induced resistance mechanisms in melanoma. Potential targets are illustrated as well as inhibitors. Vemurafenib targets BRAFV600 and trametinib targets MEK in the MAPK pathway, everolimus inhibits mTOR which is downstream of AKT and PKB, MK‐2206 is a PI3K pathway inhibitor targeting all three AKT isoforms, cetuximab and erlotinib targets the EGF receptor, sapatinib is a pan ERBB inhibitor and lastly bemcentinib selectively targets the AXL receptor.

Adaptive cellular behavior involving RTK upregulation in response to MAPK treatment is of great interest, and patient trials combining MAPK inhibitors with RTK inhibitors are ongoing (ClinicalTrials.gov; NCT02872259). However, it is important to consider the tumor heterogeneity, as well as the complexity of the melanoma signaling network. Receptor redundancy and pathway crosstalk is a major hindrance that complicates treatment tremendously.^3^, ^15^, ^16^, ^27^, ^28^ A recent study proposes that human melanoma cells display a profound transcriptional variability^8^ and that the addition of vemurafenib will induce epigenetic reprogramming in a subset of cells. During the first week of vemurafenib treatment, loss of SOX10 binding sites was observed, while 1–4 weeks of treatment revealed gain of binding sites mostly attributed to TEAD and AP‐1 activation, suggesting dedifferentiation followed by activation of novel signaling pathways conferring to stable resistance.^8^, ^29^ These observations are in agreement with Verfaille et al. in suggesting SOX10/MITF and TEAD/AP1 as master regulators of the proliferative and invasive phenotype, respectively.^9^ In accordance with this, we find that ERBB3, SOX10, and MITF often are up‐regulated in a preresistant state, while switching to an invasive phenotype leads to increased levels of AXL, NRG1 and EGFR as detected in vemurafenib‐resistant melanoma cells. To sum up our findings we have included a proposed model for stratification of our cell lines according to their transcriptional signatures (Figure 4A). In addition, we present a schematic figure (Figure 4B) showing regulators and possible targets in signal pathways involved in vemurafenib‐induced resistance.

## 3 CONCLUSION

Identification of the mechanisms behind the adaptive behavior of melanoma during progression to treatment resistance is crucial for facilitating the development of novel therapies, and for improving responses to current treatment strategies. We therefore propose that identifying expression levels of MITF/SOX10, AXL/EGFR/ERBB3 prior to initiation of treatment may contribute to predicting treatment response to MAPK inhibitors and perhaps advice on drug combination strategies in melanoma.

## 4 MATERIALS AND METHODS

### 4.1 Cell lines and culture conditions

Melanoma cell lines, WM35, WM1341, WM115, WM1366, WM45.1, WM266.4, WM983B, WM852, and WM9 cell lines were obtained from the Wistar Institute, SKMEL28, MeWo, and A375 were obtained from the American Type Culture Collection (Rockville, MD, USA), and LOXIMVI established in‐house. The immortalized melanocyte cell line Hermes 3C was purchased from the Wellcome Trust Functional Genomic cell bank.^30^ All melanoma cell lines and the Hermes 3C cell line were cultured as previously described.^13^ The cells were maintained at 37°C in a humidified atmosphere containing 5% CO_2_. Cell line identities were verified by short tandem repeat analysis and were routinely tested for Mycoplasma infections (VenorGeM, Minerva Biolabs, Berlin, Germany).

### 4.2 Transfection and RNA interference

Cells were seeded on six‐well plates and grown to 60% confluence. Cells were then transfected by RNA silencing and incubated for 72 h using siRNA directed against MITF‐M and SOX10 (Eurogentec, Seraing, Belgium), ERBB3 (Life Technologies), and AXL Silencer® Select (S1845) (Thermo Fisher Scientific). After testing several time points a 72 h incubation time was chosen to ensure an optimal reduction of our targets at the protein level. The cells were transfected with a final concentration of 25 pmol siRNA using Lipofectamine RNAiMAX (Invitrogen) as described in the manufacturer's protocol. Sequences for siRNA can be found in the Supplementary Methods. All siRNA transfections were performed in triplicate.

### 4.3 Western immunoblotting

Melanoma protein cell lysates were separated using SDS page using 4%–12% NuPAGE® Novex Bis‐Tris Midi‐ Gels (Invitrogen, Carlsbad, CA), and then transferred to a nitrocellulose membrane using iBot2 dry blotting system (Invitrogen, Carlsbad, CA). The membranes were then blocked using 5% BSA for 1 h, before incubation with primary antibody at 4°C overnight. To remove residual primary antibodies the membranes were washed for 3 × 10 min in TBS/T (20 mM Tris–HCl pH 7.5, 137 mM NaCl, and 0.1% tween20). Next, a horseradish peroxidase conjugated secondary antibody was applied for 1 h at room temperature (Dako, Glostrup, Denmark). The protein bands were visualized by chemiluminescence using SuperSignal™ West Dura Extended Duration substrate (Thermo Fisher Scientific, Waltham, MA, USA). Protein bands were visualized using the Bio‐Rad ChemiDoc™ imaging system (Bio‐Rad), and images were prepared using Adobe Photoshop CC 2017 (San Jose, CA, USA).

### 4.4 Antibodies and inhibitors

Antibodies were purchased from Cell Signaling Technology (Danvers, MA, USA); MITF (1:1000; #12590), Her3/ERBB3 XP (1:1000; #12708), AXL (1:1000 #8661), SOX10 (1:1000; #14374), Phospho‐Akt‐Serine‐473 XP® (1:2000; #4060), Phospho‐p44/42 (ERK1/2) XP® (1:2000; #4370S) and Histone H3 (1:3000; #4499) was used as a loading control. Secondary antibody against rabbit (1:5000; P0448) was purchased from Dako (Agilent Technologies, Glostrup, Denmark). The small molecular inhibitors vemurafenib (Plexxikon 4032), AZD8931 (Sapatinib) and R428 (BGB324/bemcentinib) were obtained from Selleck Chemicals (Houston, TX, USA). Recombinant human NRG1‐beta protein‐ligand was purchased from ImmunoTools GmbH (Friesoythe, Germany). Recombinant human GAS6 protein‐ligand was purchased from R&D Systems (Minneapolis, USA).

### 4.5 RNA isolation

Total RNA was isolated from the melanoma cells using the GenElute Mammalian Total RNA Miniprep Kit (Sigma‐Aldrich, Steinheim, Germany). For reverse transcription, the qScript™ cDNA Synthesis Kit (Quanta Biosciences, Gaithersburg, USA) was used. Both kits were used according to the manufacturer's manuals. Total RNA was then measured using NANODROP 2000 (Thermo Scientific).

### 4.6 Real‐time PCR

The SYBR Green system was used for Real‐time detection. 30 μl PerfeCTa™ SYBR® Green SuperMix for iQ (Quanta Biosciences, Gaithersburg, USA), 100 ng cDNA, 300 nM of each primer and nuclease‐free water were added to a final volume of 60 μl was used for each PCR reaction. The final volume of 60 μl was then split into two parallels of 25 μl and added to the PCR plate. Primers against MITF‐M, ERBB3, SOX10, AXL, GAS6, and NRG1 were ordered from Integrated DNA Technologies (IDT). Real‐time reactions were run on a CFX Connect Real‐Time PCR Detection System (Bio‐Rad) with the protocol previously described.^13^

### 4.7 Viability and proliferation assays

Cells were seeded in six‐well plates using approximately 120 000 cells per well. Cell proliferation was monitored using IncuCyte® (Essen BioScience, Hertfordshire, UK). The IncuCyte system estimates the area of the well occupied by attached cells. The viability of the cells was then measured by 3‐(4,5‐dimethylthiasol‐2‐yl)‐5‐(3‐carboxymethoxy phenyl)‐2‐(4‐sulfophenyl)‐2H‐tetrazolium (MTS) using CellTiter 96® AQ_ueous_ One Solution (Promega, Madison, WI) according to the manufacturer's protocol.

### 4.8 Statistical analysis

Statistical significance of differences between control group and RTK levels was performed using student's *t*‐test in GraphPad Prism 6. *p* < .05 were considered statistically significant. Experiments were performed in three biological replicates. For the construction of the heatmap logarithmized values (matrix)(log2 + 1) were used. Spearman's correlation was applied to calculate the correlation between MITF, ERBB3, SOX10, AXL, GAS6, and NRG1, respectively.

### 4.9 TCGA data analysis

TCGA Melanoma (TCGA‐SKCM) gene expression RNAseq files were extracted from UCSC Xena (https://xenabrowser.net/) (n = 472).^31^

All data processing was done using R software, Correlation plot was generated using the function pairs.panels( ) in the psych package. Pearson correlation analysis was used to calculate the correlation.

### 4.10 RNA expression profiling

RNA was isolated using the protocol described above. The concentration of the RNA samples was measured using the NanoDrop ND1000 spectrophotometer (Nanodrop Technologies, Delaware, USA). RNA quality was assessed using the Agilent 2100 Bioanalyzer (Agilent Technologies Inc., California, USA) and mRNA expression profiling was performed using the Illumina HumanHT‐12 v4 Expression BeadChip according to the manufacturer's protocol. Extraction of the data and quality control of the raw data was performed using Illumina's Genome studio software V2011.1. Heatmaps and clustering were performed in R.^32^

## AUTHOR CONTRIBUTIONS

**Karen‐Marie Heintz:** Data curation (supporting). **Eivind Hovig:** Funding acquisition (lead); project administration (equal); supervision (equal); writing – review and editing (equal). **Sigurd L. Bøe:** Conceptualization (equal); investigation (equal); project administration (lead); supervision (lead); writing – review and editing (equal).

## CONFLICT OF INTEREST

The authors declare that they have no competing interests.

## ETHICS STATEMENT

Not applicable.

## Supporting information


**APPENDIX S1:** Supporting Information

## Data Availability

Gene expression microarray dataset can be found in the link below: http://ous-research.no/hovig/docs/Tine/Raw_microarray_data_Melanoma_cells.zip
