# Supplementary material for: Cooperative induction of receptor tyrosine kinases contributes to adaptive MAPK drug resistance in melanoma through the PI3K pathway
Source: Cancer Rep (Hoboken). 2022 Oct 17;6(2):e1736. doi: 10.1002/cnr2.1736 (PMC9940011; doi:10.1002/cnr2.1736)

**SUPPLEMENTARY INFORMATION**

**Cooperative induction of receptor tyrosine kinases contributes to adaptive MAPK drug resistance in melanoma through the PI3K pathway**

Tine N. Alver^1,2,3^, Karen-Marie Heintz^1,3^, Eivind Hovig^1,4^, Sigurd L. Bøe^5^

^1^Department of Tumor Biology, Institute for Cancer Research, Oslo University Hospital, Oslo, Norway, ^2^Department of Cancer Genetics, Institute for Cancer Research, Oslo University Hospital, Oslo, Norway ^3^Faculty of Medicine, University of Oslo, Oslo, Norway,

^4^Department of Informatics, University of Oslo, Oslo, Norway, ^5^Department of Medical Biochemistry, Oslo University Hospital, The Norwegian Radium Hospital, Oslo, Norway

**One sentence summary:** MITF/SOX10 expression levels controls AXL/ERBB3 signaling in melanoma

**Key words:** MITF, SOX10, AXL, ERBB3, Resistance

**Corresponding author:** Tine Norman Alver, current department: Department of Cancer Genetics, Institute of Cancer Research, The Norwegian Radium Hospital, Oslo University Hospital, PO Box 4953, Nydalen, Oslo, E-mail: tinalv@rr-research.no

**Conflict of interest**: The authors declare that they have no competing interests

**SUPPLEMENTARY METHODS**

**RNA interference sequences**

MITF-M3 siRNA molecule sequence: Sense (5′- GCA-GUA-CCU-UUC-UAC-CAC-U -3′) anti sense (5′- AGU-GGU-AGA-AAG-GUA-CUG-C- 3′)

ERBB3 siRNA molecule sequence: Sense (5′- UCG-UCA-UGU-UGA-ACU-AUA-A- 3′) anti sense (5′- UUA-UAG-UUC-AAC-AUG-ACG-A -3′)

SOX10 siRNA molecule sequence: Sense (5′- GGU-CAA-GAA-GGA-ACA-GCA-G -3′) anti sense (5′- CUG-CUG-UUC-CUU-CUU-GAC-C -3′)

**Reverse Transcriptase PCR primers**

Primers against MITF-M forward (5′-CAT-TGT-TAT-GCT-GGA-AAT-GCT-AGA-3′) and reverse (5′-GC-TAA-AGT-GGT-AGA-AAG-GTA-CTG-C-3′),

SOX10 forward (5′-GAC-CAG-TAC-CCG-CAC-CTG-3′) and reverse (5′-CGC-TTG-TCA-CTT-TCG-TTC-AG-3′),

TATA-binding protein (TBP) with forward primer 5′-GCC-CGA-AAC-GCC-GAA-TAT-3′ and reverse primer (5′-CGT-GGC-TCT-CTT-ATC-CTC-ATG-A-3′)

Human acidic ribosomal phosphoprotein PO (RPLPO) forward (5′-CGC-TGC-TGA-ACA-TGC-TCA-AC-3′) and reverse(5′-TCG-AAC-ACC-TGC-TGG-ATG-AC-3′)

ERBB3 forward (5′-CTG-ATC-ACC-GGC-CTC-AAT-3′) and reverse (5′-GGA-AGA-CAT-TGA-GCT-TCT-CTG-G-3′),

AXL forward (5`-AACCAGGACGACTCCATCC-3′) and reverse

(5′-AGCTCTGACCTCGTGCAGAT-3′)

**Full length western blots**

**Figure 1E.**

Cell line: WM115

Protein: SOX10


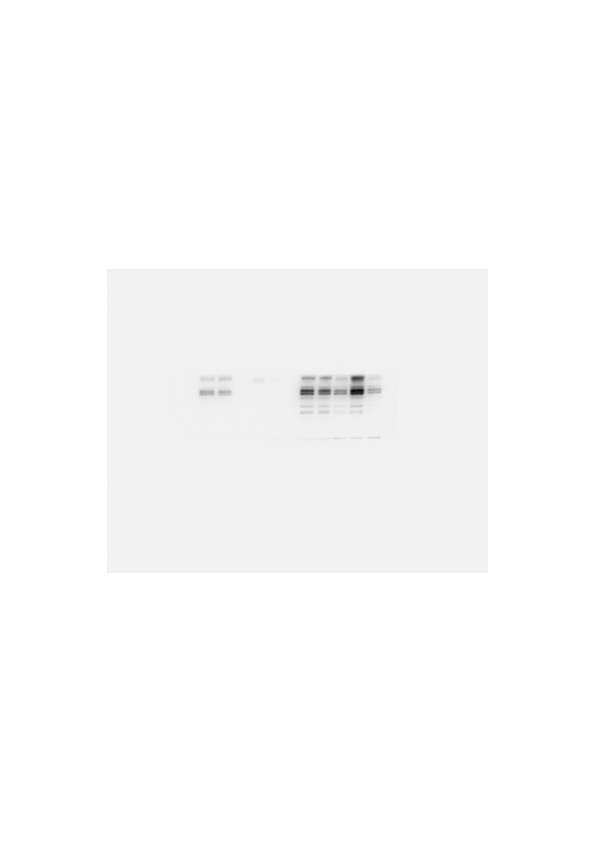


Protein: ERBB3


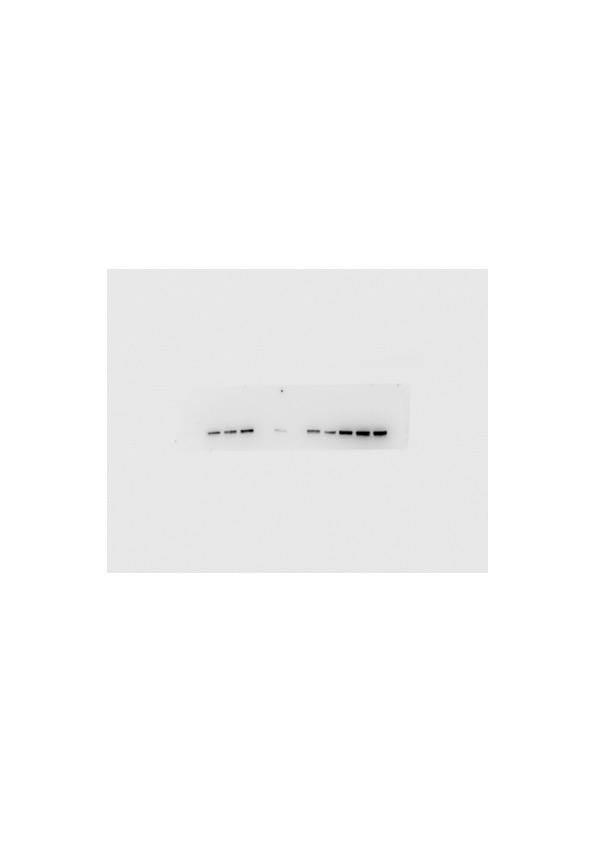


Protein: MITF


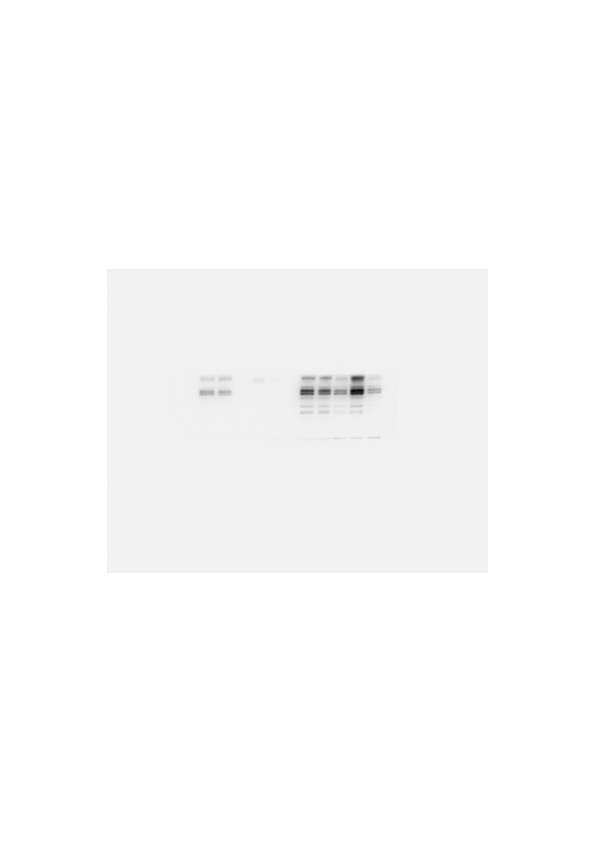


Protein: AXL


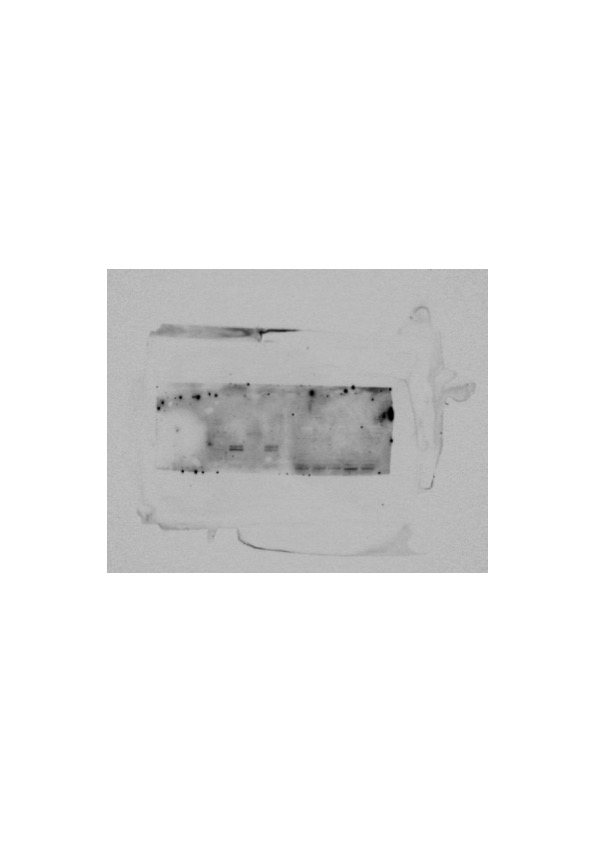


Protein: Histone H3


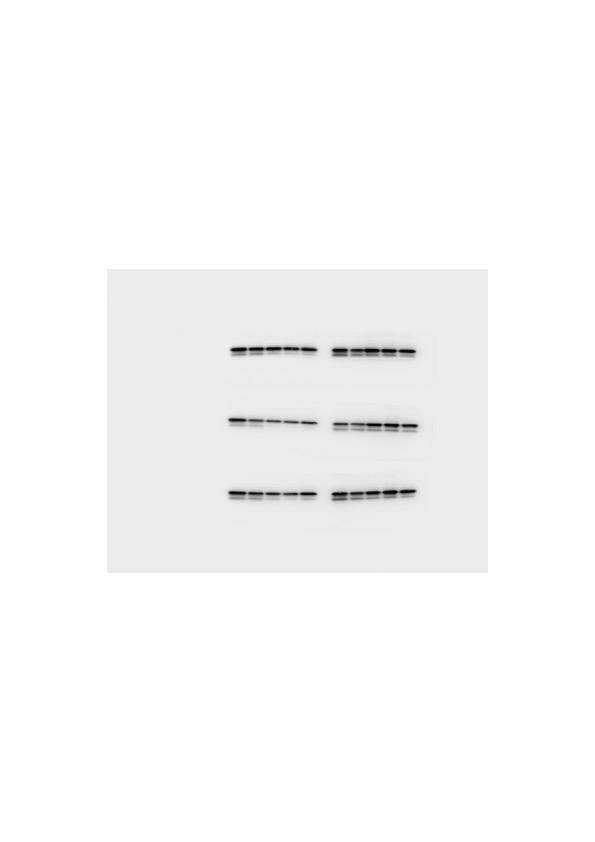


**Figure 2.**

Cell lines: WM983B/FEMXI

Protein: pAKT


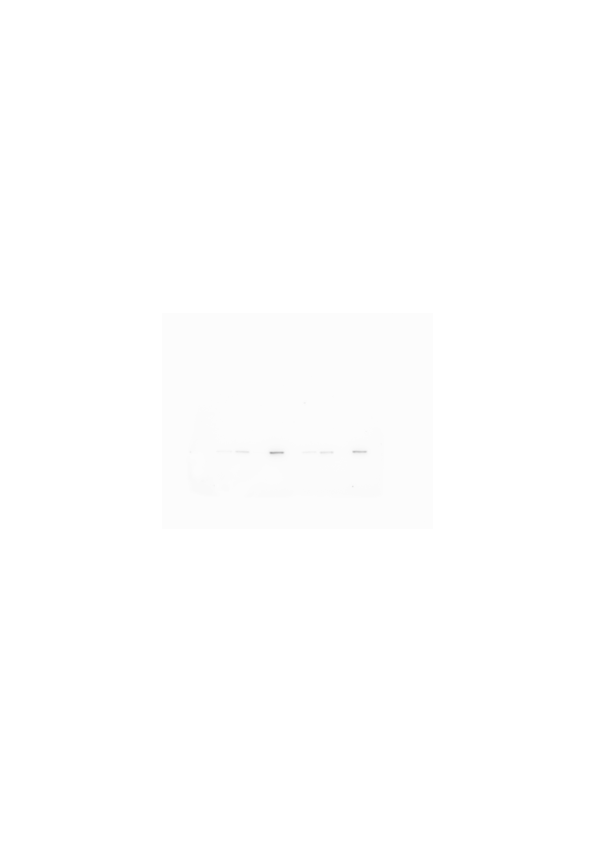


Cell line: WM983B

Protein: MITF


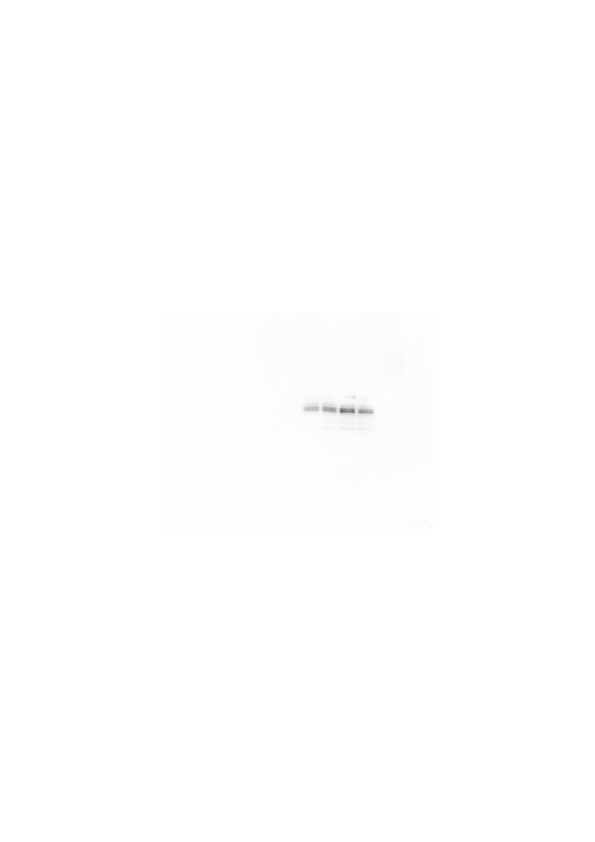


Cell line: FEMXI

Protein: MITF


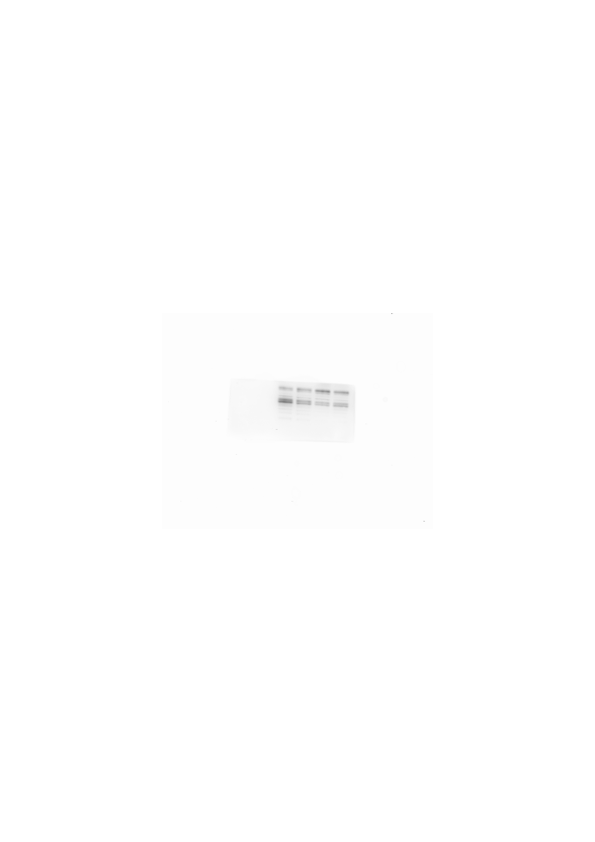


Cell line: WM983/FEMXI

Protein: ERBB3


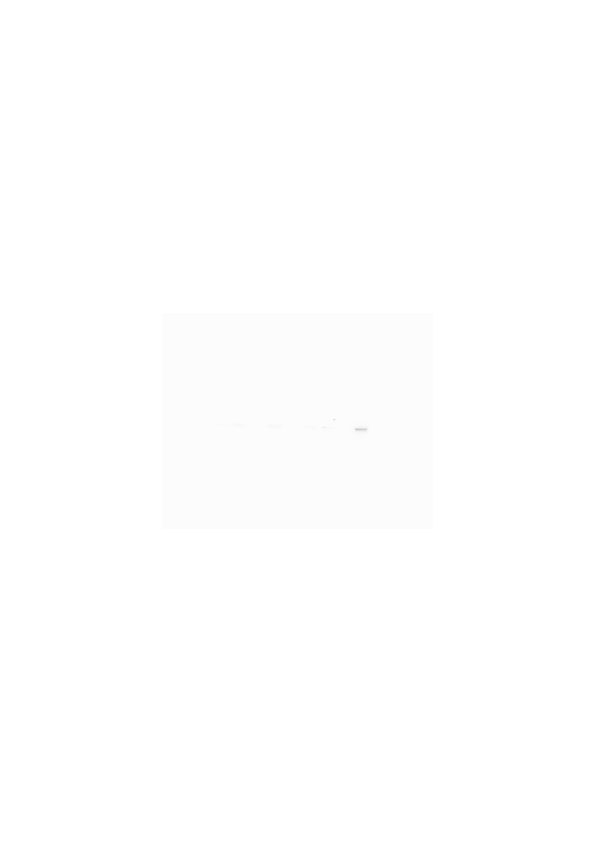


Cell line: WM983B/FEMXI

Protein: AXL


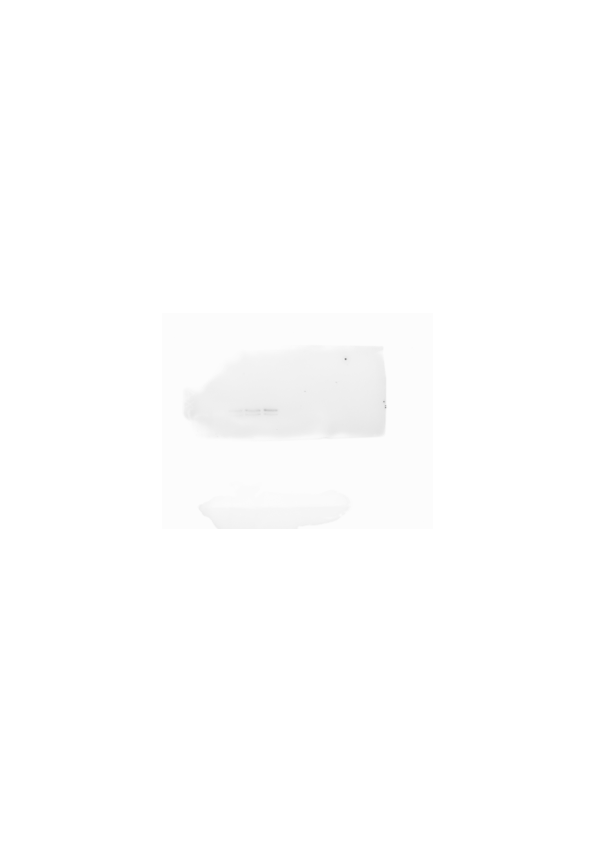


Cell line: WM983B/FEMXI

Protein: Histone H3


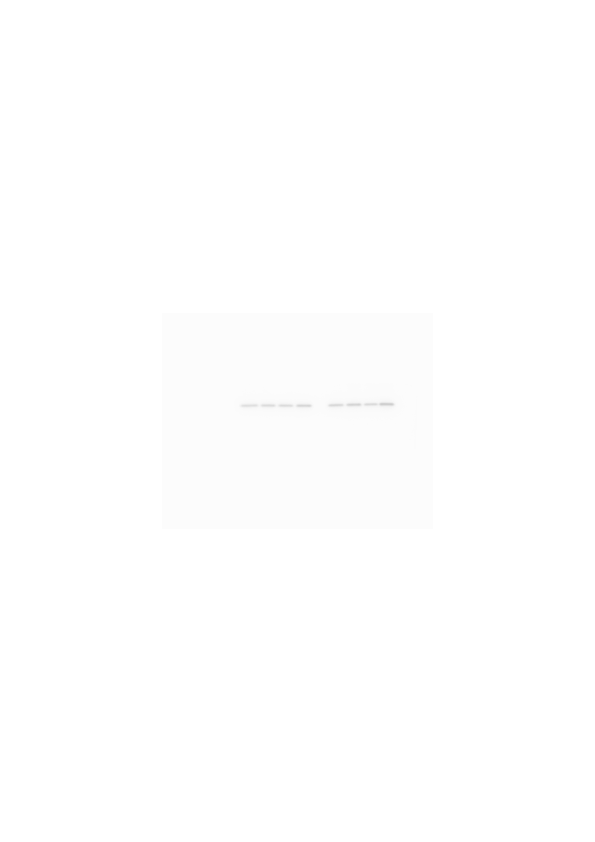


Cell line: MeWo

Protein: MITF


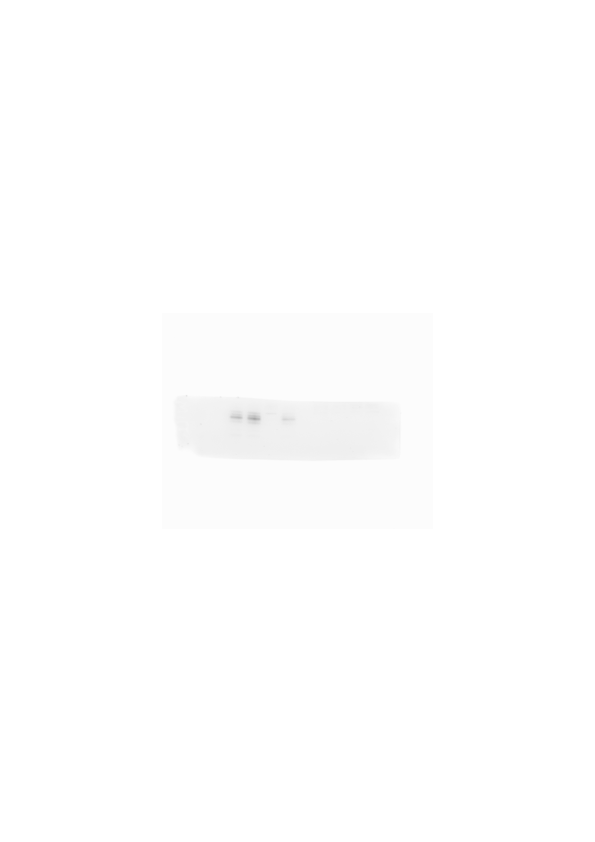


Cell line: MeWo

Protein: pAKT


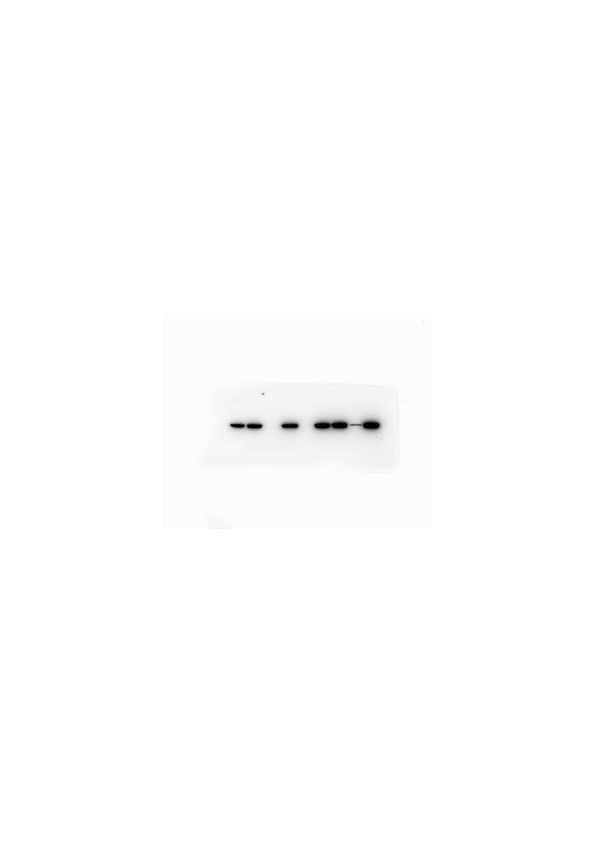


Cell line: MeWo

Protein: ERBB3


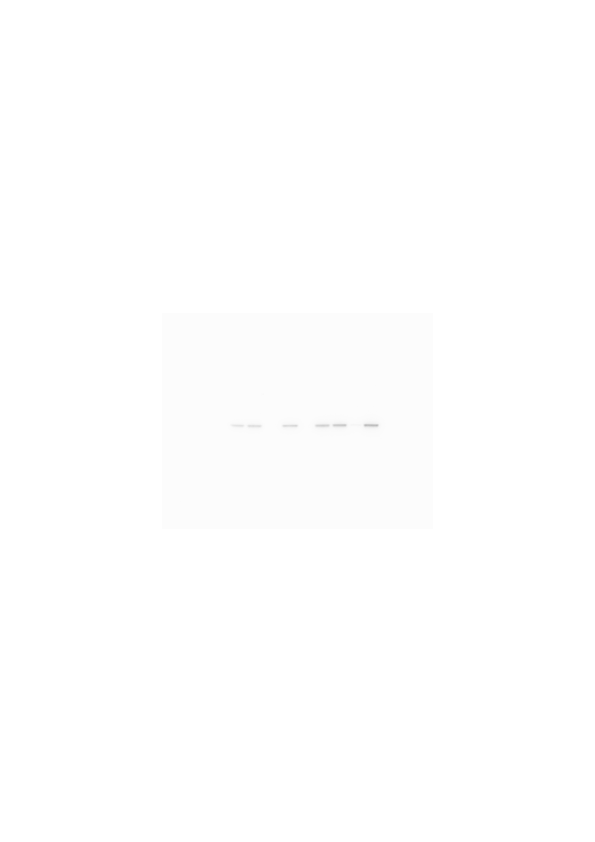


Cell line: MeWo

Protein: AXL


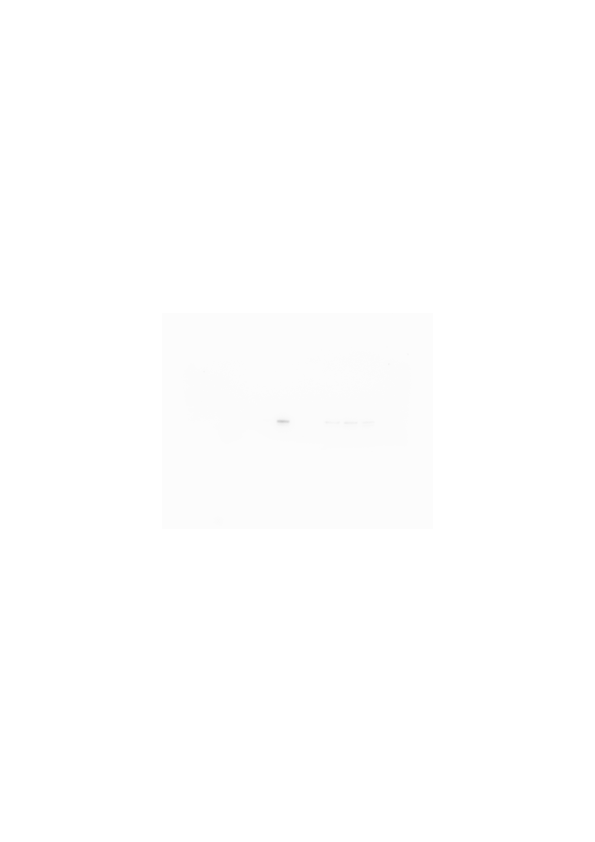


Cell line: MeWo

Protein: Histone H3


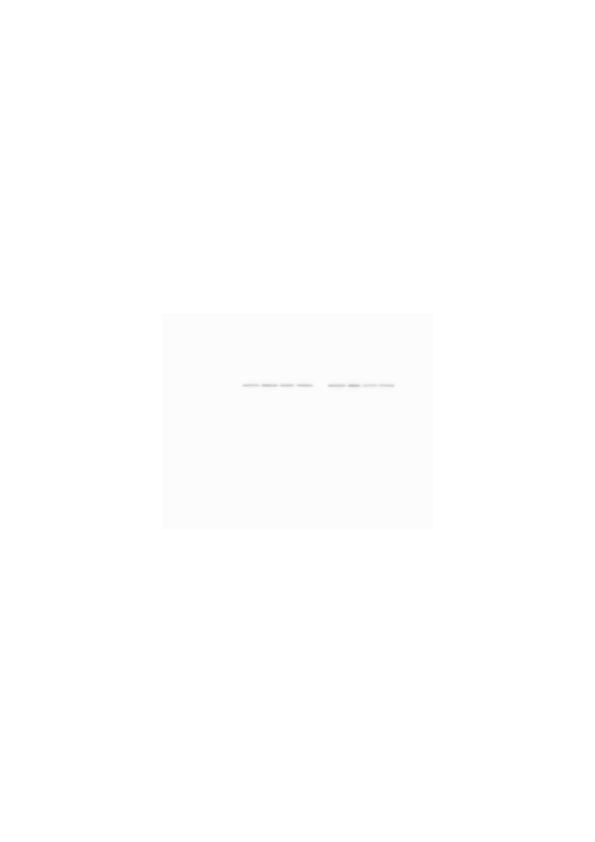


**Figure 3B.**

Cell line: A375/SKMEL28

Protein: AXL


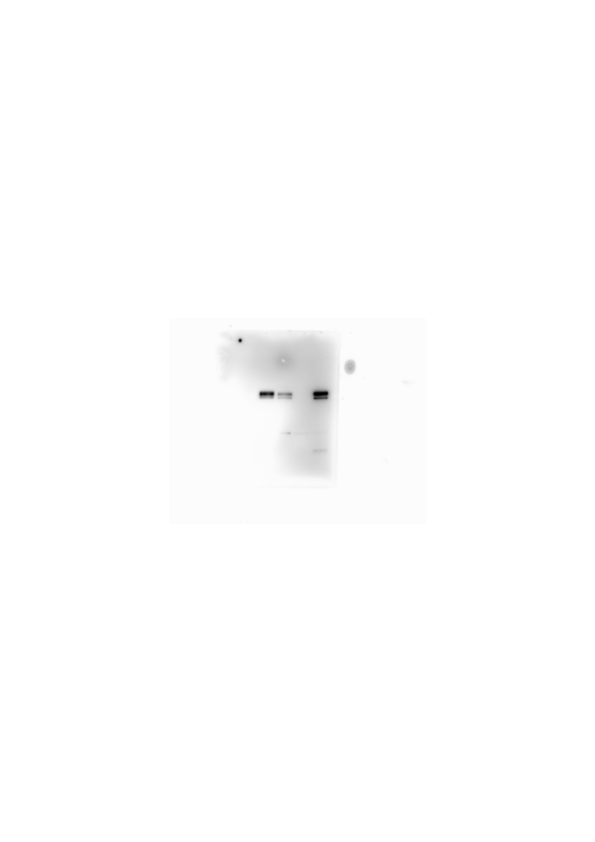


Protein: pERK


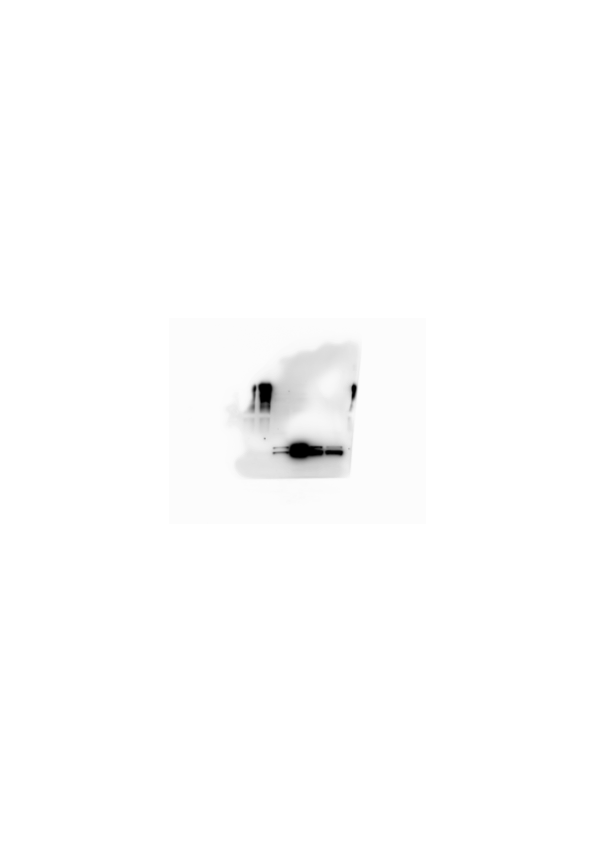


Protein: ERBB3


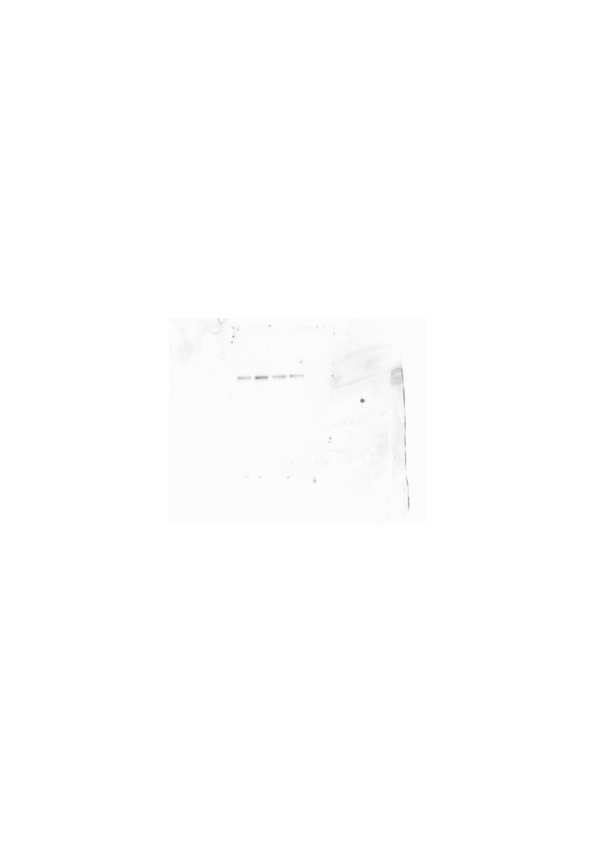


Protein: pAKT


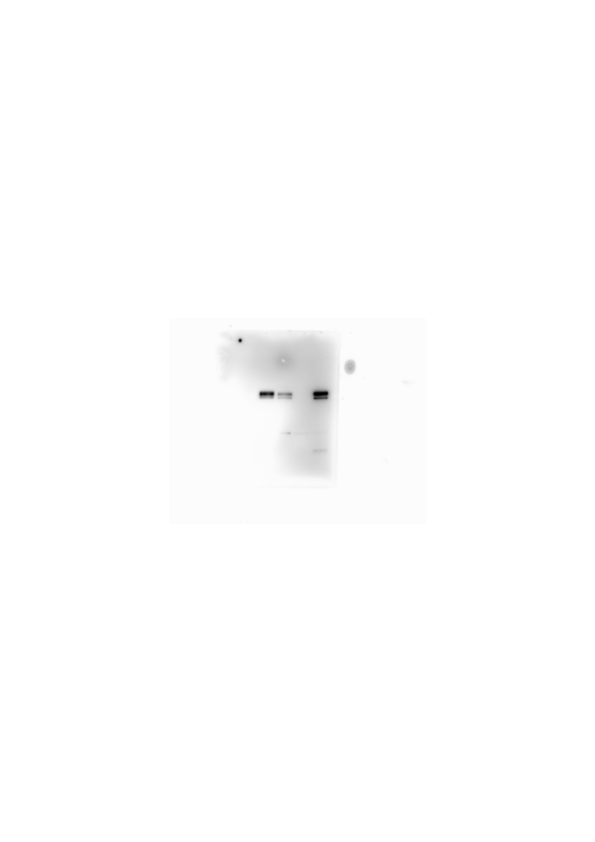


Protein: Histone H3


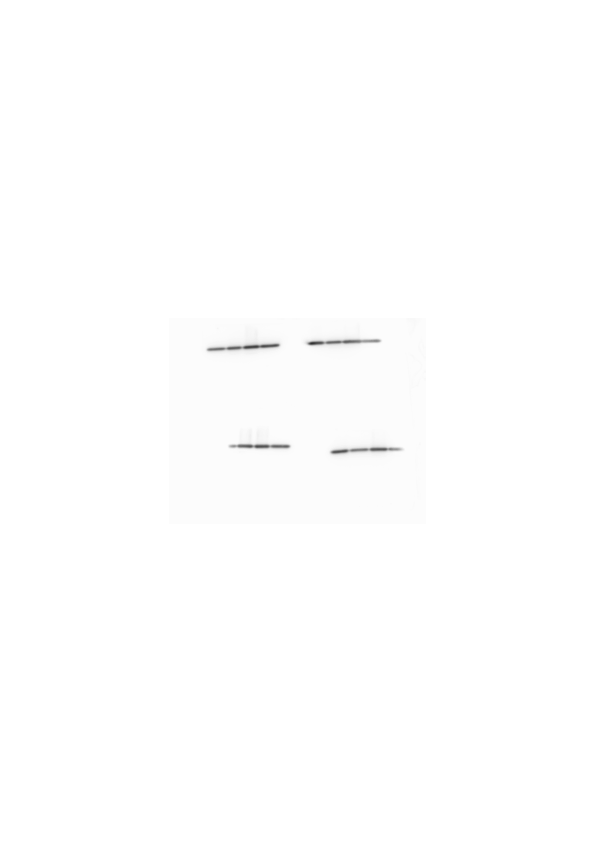

Supplement: Supplementary file 1 — APPENDIX S1: Supporting Information [file CNR2-6-e1736-s001.docx]
